# Biochemistry: Production of High-Added Value Biomolecules for Industrial Uses

**DOI:** 10.1155/2018/4012145

**Published:** 2018-02-13

**Authors:** Maha Karra-Chaabouni, Mohamed Trigui, María M. Yust, Mireille K. Awad, Pedro J. García-Moreno

**Affiliations:** ^1^Preparatory Institute for Engineering Studies, University of Sfax, Sfax, Tunisia; ^2^Instituto de la Grasa-CSIC, Sevilla, Spain; ^3^Saint-Joseph University, Beirut, Lebanon; ^4^National Food Institute, Technical University of Denmark, Kongens Lyngby, Denmark

Natural resources (plant, microorganisms, and algae) constitute a renewable reservoir of high-added value molecules used in various fields such as health, food, pharmaceuticals, and cosmetics. These molecules obtained by extraction or bioconversion are considered natural and have gained an increasing interest at the expense of synthetic products. This is due to the fact that consumers are raising awareness towards the benefits of natural products particularly in food, beverages, and medicines. Moreover, the development of different bioprocesses, particularly techniques for biomolecules extraction and bioconversion (e.g., maceration, supercritical-fluid extraction, fermentation, and enzyme catalysis), allows the discovery of novel bioactive compounds which can be potentially used as drugs or for the fortification of foods.

This special issue contains five papers related to the production of high-added value biomolecules from natural resources and their use in industrial applications. An overview of the research works published in this special issue is given below.

Microorganisms continuously provide new bioactive compounds which are used for the development of novel drugs for the treatment of human, animal, and plant diseases, especially the production of antibiotics more effective against resistant microbes. In this special issue two papers investigate the capacity of microorganisms isolated from extreme conditions to produce active biomolecules.* In the paper* entitled “Antagonistic Properties of Some Halophilic Thermoactinomycetes Isolated from Superficial Sediment of a Solar Saltern and Production of Cyclic Antimicrobial Peptides by the Novel Isolate* Paludifilum halophilum,*” D. F. Dammak et al. have isolated halophilic actinomycetes from a concentrator and crystallizer solar saltern ponds and explored their potential to produce drugs against agricultural and human pathogens.* In the paper* entitled “The Potential of a Brown Microalga Cultivated in High Salt Medium for the Production of High-Value Compounds,” S. Boukhris et al. investigated the physicochemical properties of bioactive compounds produced from* Amphora *sp. (Bacillariophyceae) cultivated in a hypersaline medium. The fatty acids profile and biological activities (antioxidant and antibacterial) of the ethanolic extract of* Amorpha *sp. were also determined.

Phytochemicals extracted from plants are a rich source of bioactive molecules including phenolics, vitamins, and flavonoids. These molecules have been recognized as the most promising compounds for the development of medicines used in several pharmacological activities (e.g., anti-inflammation, antimicrobial, antihypertension). This is the subject of the following three papers published in this special issue.* In the paper* entitled “Kinetics of Tyrosinase Inhibitory Activity Using* Vitis vinifera *Leaf Extracts,” Y.-S. Lin et al. studied the tyrosinase inhibitory activity of red vine leaf extract (RVLE) containing gallic acid, chlorogenic acid, epicatechin, rutin, and resveratrol, which are effective compounds for skin hyperpigmentation. The authors reported that RVLE had an effective tyrosinase inhibitory activity and can be used as a whitening agent for cosmetic formulations in the future.* In the paper* entitled “Nutritional Composition and Phytochemical, Antioxidative, and Antifungal Activities of* Pergularia tomentosa *L.”, I. Lahmar et al. evaluated the antioxidant properties of extracts from four different organs (roots, stems, leaves, and fruits) of a medicinal Tunisian plant,* Pergularia tomentosa *L. In addition, this work showed that stem and fruit extracts exhibit an antifungal activity against* Fusarium oxysporum *f. sp.* lycopersici, *which could become an alternative to synthetic fungicide.* In the paper* entitled “*Citrus limon *from Tunisia: Phytochemical and Physicochemical Properties and Biological Activities” M. Makni et al. realized quantitative and qualitative characterizations of the zest and the flesh of lemon* (Citrus limon)*. In order to valorize the pharmacological uses of lemon, the authors evaluated its biological activities (antioxidant, antibacterial, antifungal, and antiproliferative activities).

We hope that this special issue provides to the readers with valuable and useful knowledge contributing to the scientific research progress in the biology field.

